# Supplementary material for: The impact of insect herbivory on biogeochemical cycling in broadleaved forests varies with temperature
Source: Nat Commun. 2024 Jul 17;15:6011. doi: 10.1038/s41467-024-50245-9 (PMC11254921; doi:10.1038/s41467-024-50245-9)
Supplement: Supplementary file 1 — Supplementary Information [file 41467_2024_50245_MOESM1_ESM.pdf]

Supplementary Information for "Temperature mediates insect herbivore impact on element cycling in broadleaved forests" (Hwang et al. 2024)

Supplementary Table 1. Acronyms and equations

Acronyms (Fig. 1) and equations used for herbivory-related calculations are patterned after Metcalfe et al[1].

Description	Acronym	Unit	Equation
Element C, N, P, or Si	E	NA	NA
Green leaf concentration of E	F_E	proportion	NA
Leaf litter concentration of E	B_E	proportion	NA
Foliar production	FP	g m^{-2} y^{-1}	L_H / (1 - H)
Herbivory as proportion of leaf litter area removed (folivory)	H	proportion	NA
E consumed by insect folivores (gross input of E by insect folivores)	H_cE	g m^{-2} y^{-1}	FP × F_E × H
Additional E input from insect folivores due to green leaf consumption before resorption (net input of E by insect folivores)	H_iE	g m^{-2} y^{-1}	L_{EH} + H_C - L_E
Foliar input of E to soil without H (litter-mediated input of E)	L_E	g m^{-2} y^{-1}	(FP × F_E) × (1 - RE_E)
Foliar input of E to soil with H	L_{EH}	g m^{-2} y^{-1}	L_H × L_E × (1 - RE_E)
Annual broadleaved litterfall with H	L_H	g m^{-2} y^{-1}	Total annual dry weight of leaf litter / area of trap
Global mass correction factor due to leaf senescence (Vergutz et al., 2012)	Q	proportion	NA
Resorption of E	RE_E	proportion	1 - (L_E / F_E × Q)
Soil concentration of E	S_E	proportion	NA

Supplementary Fig. 1. Site distribution

Green shading represents forest cover[2]. Circles indicate locations of mature, undisturbed broadleaved boreal (blue), temperate (black), and tropical (orange) forest stands included in this study. Altogether, the network was comprised of 74 forest plots in 40 sites. Base map adapted from Google Maps.

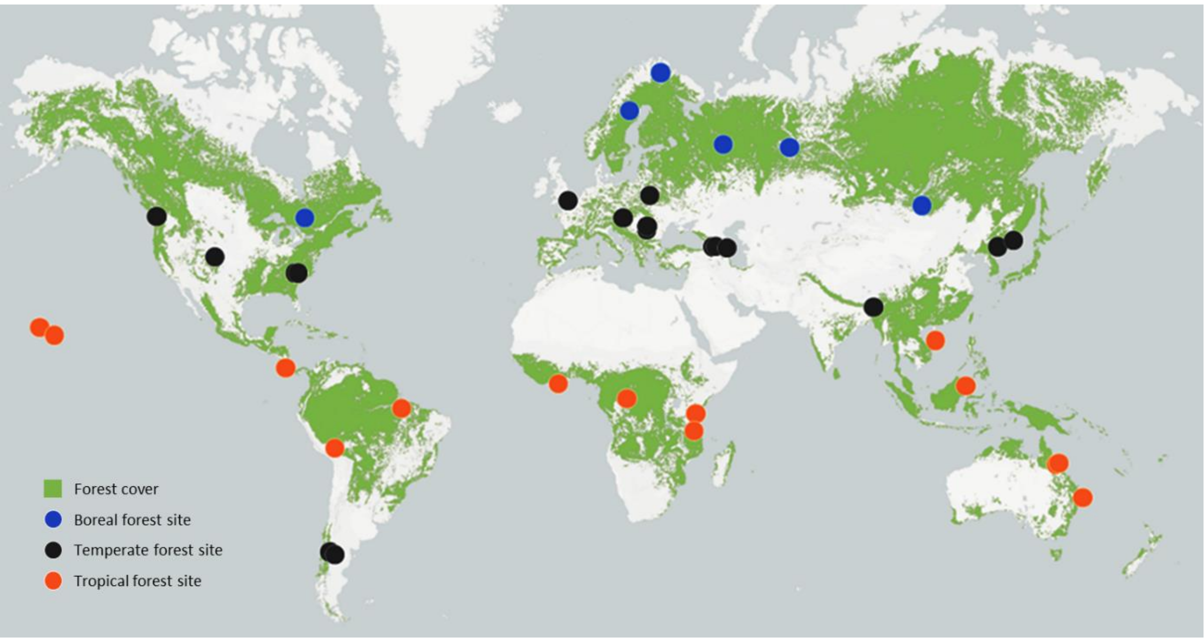

Supplementary Table 2. Global means, minimums, and maximums across broadleaved forest plots

Global means, minimums, and maximums of ecological characteristics across all plots and gradients with standard error (74 plots, 9-25 litter traps per plot). Source data are provided as a Source Data file.

Variable	Unit	Mean	Minimum	Maximum
Mean annual temperature (MAT)	°C	12.0	-1.4	26.9
Mean annual precipitation (MAP)	mm	1792.1	434	5500
Potential evapotranspiration (PET)	mm	974.5	246.4	1648.9
Water stress ratio (PET/MAP)	ratio	0.6	0.2	1.3
Latitude	decimal degrees	17.6	-41.2	69.8
Absolute latitude	decimal degrees	31.5	1.2	69.8
Elevation	m	844.8	7	2981
Foliar production (FP)	$g\ m^{-2}\ y^{-1}$	381.1 ± 6.6	43.5 ± 3.9	1174.0 ± 64.8
Foliar herbivory (H)	% leaf area removed	4.12 ± 0.97	0.63 ± 0.08	13.58 ± 0.77
Solar radiation (SR)	$MWh\ m^{-2}$	1.30 ± 0.04	0.73	1.98
Soil C (S_C)	% dry weight	8.2 ± 0.8	0.8	27.2
Soil N (S_N)	% dry weight	0.49 ± 0.05	0.04	1.81
Soil P (S_P)	% dry weight	0.0771 ± 0.0076	0.0004	0.2785
Soil Si (S_{Si})	% dry weight	2.6 ± 0.5	0.1	24.7
Green leaf C (F_C)	% dry weight	47.8 ± 0.3	42.6	55.7
Green leaf N (F_N)	% dry weight	1.91 ± 0.06	0.6	3.1
Green leaf P (F_P)	% dry weight	0.13 ± 0.01	0.02	0.24
Green leaf Si (F_{Si})	% dry weight	0.7 ± 0.1	0.1	7.5
Litter C (B_C)	% dry weight	49.1 ± 0.3	41.7	55.4
Litter N (B_N)	% dry weight	1.14 ± 0.04	0.41	2.03
Litter P (B_P)	% dry weight	0.067 ± 0.005	0.009	0.233
Litter Si (B_{Si})	% dry weight	1.1 ± 0.1	0.1	4.6
C resorption efficiency (RE_C)	%	19.7 ± 0.4	9.3	32.5
N resorption efficiency (RE_N)	%	51.5 ± 1.6	15.7	72.9
P resorption efficiency (RE_P)	%	61.5 ± 1.4	22.9	81.7
Litter flux of C (L_C)	$g\ m^{-2}\ y^{-1}$	179.3 ± 3.05	21.7	555.9
Litter flux of N (L_N)	$g\ m^{-2}\ y^{-1}$	4.4 ± 0.1	0.3	14.6
Litter flux of P (L_P)	$g\ m^{-2}\ y^{-1}$	0.215 ± 0.04	0.01	0.73
Litter flux of Si (L_{Si})	$g\ m^{-2}\ y^{-1}$	4.92 ± 0.21	0.06	38.31
H_c of C (H_cC)	$g\ m^{-2}\ y^{-1}$	9.1 ± 0.4	0.2 ± 0.1	71.7 ± 44.2
H_c of N (H_cN)	$g\ m^{-2}\ y^{-1}$	0.36 ± 0.01	0.0071 ± 0.0001	2.0 ± 1.2
H_c of P (H_cP)	$g\ m^{-2}\ y^{-1}$	0.022 ± 0.001	0.0007 ± 0.0001	0.15 ± 0.01
H_c of Si (H_cSi)	$g\ m^{-2}\ y^{-1}$	0.15 ± 0.01	0.0005 ± 0.0001	1.9 ± 0.2
H_i of C (H_iC)	$g\ m^{-2}\ y^{-1}$	1.7 ± 0.1	0.03 ± 0.01	10.9 ± 0.5
H_i of N (H_iN)	$g\ m^{-2}\ y^{-1}$	0.17 ± 0.02	0.005 ± 0.001	0.8 ± 0.1
H_i of P (H_iP)	$g\ m^{-2}\ y^{-1}$	0.014 ± 0.001	0.0004 ± 0.0001	0.10 ± 0.01

Supplementary Table 3. Ecological characteristics of broadleaved forests across latitude zones. Means of ecological characteristics across tropical (33), temperate (32) and boreal (9) forests with standard errors. Different letters following means indicate significant differences based on two-sided Kruskal-Wallis and Dunn-Bonferroni post-hoc tests (95% CI). Source data are provided as a Source Data file.

Variable	Units	Tropical	Temperate	Boreal	X^2	DF	*P*
Mean annual temperature (MAT)	°C	19.47 ± 0.51[a]	7.76 ± 0.51[b]	-0.28 ± 0.33[c]	58.02	2	< 0.001
Mean annual precipitation (MAP)	mm	2426.91 ± 129.75[a]	1388.81 ± 129.75[b]	559.44 ± 45.31[c]	34.77	2	< 0.001
Potential evapotranspiration (PET)	mm	1317.09 ± 17.78[a]	775.85 ± 17.78[b]	424.76 ± 51.32[c]	59.89	2	< 0.001
Dryness or climate ratio	ratio	0.63 ± 0.05	0.66 ± 0.05	0.59 ± 0.05	0.28	2	0.869
Foliar biomass production (FP)	$g\ m^{-2}\ y^{-1}$	513.0 ± 10.2[a]	321.6 ± 7.3[b]	231.9 ± 17.5[c]	32.05	2	< 0.001
Foliar herbivory (H)	% leaf area	5.4 ± 0.2[a]	3.3 ± 0.1[b]	2.4 ± 0.2[b]	8.49	2	0.014
Soil C	% dry weight	10.33 ± 0.56	7.31 ± 0.56	4.05 ± 0.92	3.86	2	0.145
Soil C : N	ratio	17.74 ± 0.67[ab]	15.47 ± 0.67[b]	22.19 ± 2.31[a]	10.73	2	0.005
Soil C : P	ratio	275.74 ± 8.25[a]	107.83 ± 8.25[b]	106.71 ± 28.1[b]	6.12	2	0.047*
Soil Si	% dry weight	2.47 ± 0.87	3.13 ± 0.87	1.24 ± 0.9	3.72	2	0.155
Foliar C	% dry weight	48.38 ± 0.41	47.69 ± 0.41	47.51 ± 0.66	1.29	2	0.525
Foliar N	% dry weight	1.64 ± 0.09[a]	0.021 ± 0.001[b]	0.022 ± 0.001[b]	11.73	2	0.003
Foliar P	% dry weight	0.10 ± 0.01[a]	0.14 ± 0.01[b]	0.18 ± 0.01[c]	26.80	2	< 0.001
Foliar Si	% dry weight	0.47 ± 0.25	1.01 ± 0.25	0.55 ± 0.16	5.27	2	0.072
Litter C	% dry weight	50.08 ± 0.33[a]	48.7 ± 0.33[b]	48.77 ± 0.58[ab]	11.93	2	0.003
Litter N	% dry weight	1.12 ± 0.06	1.19 ± 0.06	1.07 ± 0.12	0.23	2	0.893
Litter P	% dry weight	0.04 ± 0.01[a]	0.08 ± 0.01[b]	0.10 ± 0.02[b]	36.18	2	< 0.001
Litter Si	% dry weight	1.00 ± 0.18[a]	1.36 ± 0.18[b]	0.69 ± 0.21[ac]	6.96	2	0.031*
C resorption	proportion	0.19 ± 0.01	0.20 ± 0.01	0.20 ± 0.01	1.20	2	0.549
N resorption	proportion	0.47 ± 0.03[a]	0.53 ± 0.03[ab]	0.62 ± 0.03[b]	12.07	2	0.002
P resorption	proportion	0.67 ± 0.02[a]	0.58 ± 0.02[b]	0.55 ± 0.05[b]	14.05	2	< 0.001
Gross insect-mediated C flux	$g\ m^{-2}\ y^{-1}$	15.87 ± 0.90[a]	5.41 ± 0.90[b]	1.65 ± 0.58[b]	24.37	2	< 0.001
Gross insect-mediated N flux	$g\ m^{-2}\ y^{-1}$	0.600 ± 0.038[a]	0.231 ± 0.038[b]	0.082 ± 0.029[b]	14.21	2	< 0.001
Gross insect-mediated P flux (H_cP)	$g\ m^{-2}\ y^{-1}$	0.034 ± 0.003[a]	0.016 ± 0.003[b]	0.006 ± 0.002[b]	12.17	2	0.003
Gross insect-mediated Si flux	$g\ m^{-2}\ y^{-1}$	0.178 ± 0.076[a]	0.178 ± 0.76[ab]	0.029 ± 0.012[b]	7.04	2	0.030
H_cC : Litter C	ratio	0.055 ± 0.007[a]	0.035 ± 0.005[b]	0.026 ± 0.009[b]	9.62	2	0.008
H_cN : Litter N	ratio	0.084 ± 0.010	0.061 ± 0.009	0.051 ± 0.014	5.68	2	0.058
H_cP : Litter P	ratio	0.136 ± 0.016[a]	0.072 ± 0.013[b]	0.042 ± 0.008[b]	17.68	2	< 0.001
H_cSi : Litter Si	ratio	0.035 ± 0.004	0.031 ± 0.008	0.024 ± 0.010	5.83	2	0.054

* No significant differences between latitude zones in Dunn test after Bonferroni-Holm adjustment.

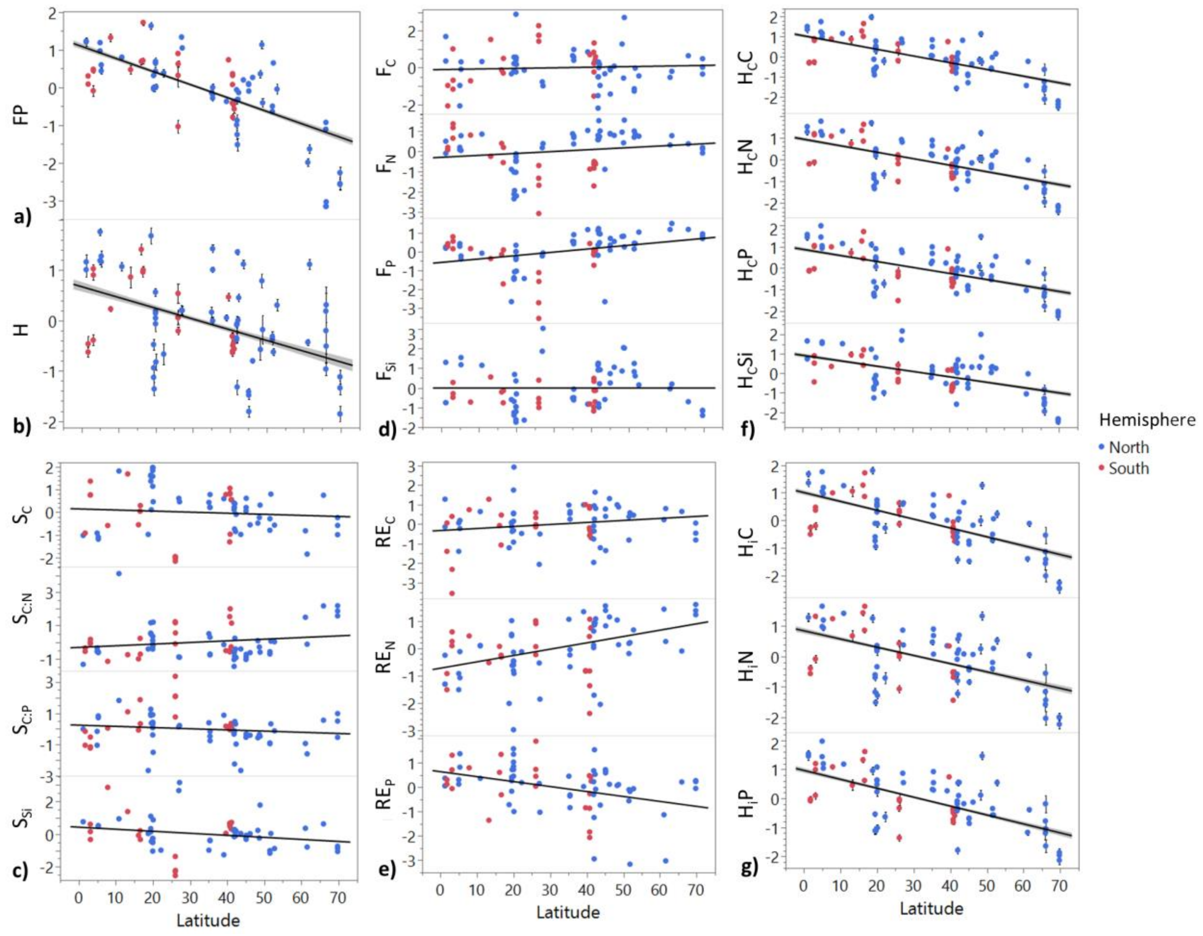

Supplementary Fig. 2. Potential explanatory variables for insect-mediated element fluxes in broadleaved forests along latitude gradient. Insect-mediated element fluxes and potential explanatory variables along a global latitudinal gradient. a) Foliar biomass production (FP, g m^{-2} y^{-1}), b) foliar herbivory rate (H, % leaf area removed y^{-1}), c) soil nutrients or stoichiometry (S_C, $S_{C:N}$, $S_{C:P}$, S_{Si}; %), d) foliar concentration of element E (F_E, %), e) resorption efficiency of E (RE_E, %), f) gross insect-mediated element flux (H_c, g m^{-2} y^{-1}), and g) net insect-mediated element flux (H_i, g m^{-2} y^{-1}) plotted against absolute latitude. Response variables were log and logit transformed as necessary and (*Z*) standardized before regressing. Blue circles represent forests in the Northern Hemisphere, red circles represent forests in the Southern Hemisphere. Circles for a), b), f) and g) depict means within a forest plot (74 plots), bars represent ± 1 SE (9-25 traps per plot), and lines represent best-fitted lines. Source data are provided as a Source Data file.

Supplementary Table 4. Relative contributions of abiotic variables on insect-mediated element fluxes in broadleaved forests globally

Mean annual temperature (MAT, °C), dryness ratio (potential evaporation/mean annual precipitation), and soil element concentration or stoichiometric ratio (C, C:N, C:P, Si) relative contributions to gross (H_c, g m^{-2} y^{-1}) and net (H_i, g m^{-2} y^{-1}) insect herbivore element fluxes. Linear mixed-effect modeling results are based on log and logit transformations of variables as appropriate, followed by z standardization. Site:plot was set as a random factor. Coefficients (COEF) indicate direction and magnitude of change, and the lower and upper ends of the 95% confidence intervals (CI) describe the uncertainty surrounding the estimates. Source data are provided as a Source Data file.

	MAT		PET/MAP		Soil C, C:N, C:P, or Si	
Flux	COEF	CI	COEF	CI	COEF	CI
H_cC	0.59*	0.42, 0.77	0.04	-0.15, 0.21	0.09	-0.09, 0.27
H_cN	0.44*	0.27, 0.62	0.05	-0.12, 0.22	-0.35*	-0.53, -0.19
H_cP	0.51*	0.32, 0.71	0.01	-0.18, 0.19	-0.29*	-0.47, -0.11
H_cSi	0.36*	0.15, 0.56	0.07	-0.10, 0.24	0.29*	0.10, 0.48
H_iC	0.59*	0.39, 0.77	0.05	-0.13, 0.26	0.09	-0.09, 0.28
H_iN	0.38*	0.21, 0.56	0.12	-0.05, 0.28	-0.31*	-0.48, -0.14
H_iP	0.52*	0.34, 0.71	-0.00	-0.18, 0.18	-0.22*	-0.41, -0.05

*Indicates significant explanatory power after 1000 bootstrapping simulations (95% CIs do not overlap with zero).

Supplementary Table 5. Relative contributions of abiotic variables on insect-mediated element fluxes in broadleaved forests within latitude zones

Relative contributions of mean annual temperature (MAT), dryness (potential evapotranspiration/mean annual precipitation), and soil nutrient content to gross insect-mediated nitrogen (H_cN) and phosphorus (H_cP) fluxes in tropical (33 plots), temperate (32 plots) and boreal forests (9 plots). Linear mixed-effect modeling results are based on log and logit transformations of variables as appropriate, followed by z standardization. Site was set as a random factor. Coefficients (COEF) indicate direction and magnitude of change and the lower and upper ends of the 95% confidence intervals (CI) describe the uncertainty surrounding the estimates. Source data are provided as a Source Data file.

Tropical	Abiotic variable	COEF	CI
H_cN	MAT*	0.60	0.28, 0.93
	PET/MAP*	0.13	0.03, 0.22
	Soil C:N*	-0.31	-0.46, -0.16
H_cP	MAT*	0.40	0.04, 0.75
	PET/MAP*	0.13	0.03, 0.24
	Soil C:P*	-0.33	-0.43, -0.25
Temperate			
H_cN	MAT	-0.18	-0.43, 0.10
	PET/MAP*	-0.15	-0.26, -0.05
	Soil C:N*	-0.18	-0.27, -0.08
H_cP	MAT	0.28	-0.004, 0.539
	PET/MAP	0.01	-0.08, 0.11
	Soil C:P*	0.22	0.12, 0.31
Boreal			
H_cN	MAT*	3.22	0.67, 5.84
	PET/MAP	-0.09	-1.18, 0.90
	Soil C:N	-0.27	-0.64, 0.13
H_cP	MAT	3.48	-1.46, 7.68
	PET/MAP	-0.07	-1.36, 1.36
	Soil C:P	-0.11	-0.47, 0.25

*Indicates significant explanatory power after 1000 bootstrapping simulations (95% CIs do not overlap with zero).

Supplementary Table 6. Relative contributions of biotic variables to insect-mediated element fluxes in broadleaved forests globally

Foliar biomass production (FP, g m^{-2} y^{-1}), foliar herbivory rate (H, % leaf area removed y^{-1}), green leaf element concentration (FE_E, %), and resorption efficiency (RE_E, %) relative contributions to gross (H_c) and net (H_i) insect herbivore element fluxes. Linear mixed-effect modeling results are based on log and logit transformations of variables as appropriate, followed by z standardization. Site:plot was set as a random factor. Coefficients (COEF) indicate direction and magnitude of change, and the lower and upper ends of the 95% confidence intervals (CI) describe the uncertainty surrounding the estimates. Source data are provided as a Source Data file.

	Foliar biomass production		Herbivory rate		Foliar element concentration		Resorption efficiency	
Flux	COEF	CI	COEF	CI	COEF	CI	COEF	CI
H_cC	0.57	0.571, 0.573	0.64	0.644, 0.646	0.04	0.035, 0.039	NA	NA
H_cN	0.58	0.547, 0.549	0.66	0.616, 0.618	0.35	0.245, 0.249	NA	NA
H_cP	0.58	0.582, 0.585	0.66	0.656, 0.658	0.35	0.347, 0.352	NA	NA
H_cSi	0.44	0.434, 0.436	0.49	0.489, 0.491	0.44	0.527, 0.530	NA	NA
H_iC	0.57	0.571, 0.574	0.65	0.644, 0.646	0.37	0.139, 0.145	0.14	0.139, 0.145
H_iN	0.57	0.572, 0.574	0.65	0.644, 0.647	0.25	0.246, 0.264	0.23	0.222, 0.238
H_iP	0.56	0.562, 0.565	0.63	0.634, 0.636	0.37	0.365, 0.383	0.21	0.209, 0.221

Supplementary references

[1]Metcalfe, D. B., Asner, G. P., Martin, R. E., Silva Espejo, J. E., Huasco, W. H., Farfán Amézquita, F. F., ... & Malhi, Y. Herbivory makes major contributions to ecosystem carbon and nutrient cycling in tropical forests. *Ecol. Lett.* **17**, 324-332 (2014).

[2]Buchhorn, M. et al. Copernicus Global Land Service: Land Cover 100m: Collection 3: epoch 2019: Globe (Version V3.0.1) [Data set]. *Zenodo* https://doi.org/10.5281/zenodo.3939050 (2020).
