# Supplementary material for: The impact of insect herbivory on biogeochemical cycling in broadleaved forests varies with temperature
Source: Nat Commun. 2024 Jul 17;15:6011. doi: 10.1038/s41467-024-50245-9 (PMC11254921; doi:10.1038/s41467-024-50245-9)
Supplement: Supplementary file 3 — Description of Additional Supplementary Files [file 41467_2024_50245_MOESM3_ESM.pdf]

Description of Additional Supplementary Files

Supplementary Data 1: Site descriptions
