# Supplementary material for: The impact of insect herbivory on biogeochemical cycling in broadleaved forests varies with temperature
Source: Nat Commun. 2024 Jul 17;15:6011. doi: 10.1038/s41467-024-50245-9 (PMC11254921; doi:10.1038/s41467-024-50245-9)
Supplement: Supplementary file 5 — Reporting Summary [file 41467_2024_50245_MOESM5_ESM.pdf]

nature portfolio

Corresponding author(s): Bernice Hwang

Last updated by author(s): May 14, 2024

Reporting Summary

Nature Portfolio wishes to improve the reproducibility of the work that we publish. This form provides structure for consistency and transparency in reporting. For further information on Nature Portfolio policies, see our Editorial Policies and the Editorial Policy Checklist.

Statistics

For all statistical analyses, confirm that the following items are present in the figure legend, table legend, main text, or Methods section.

n/a	Confirmed	
[ ]	[x]	The exact sample size (*n*) for each experimental group/condition, given as a discrete number and unit of measurement
[ ]	[x]	A statement on whether measurements were taken from distinct samples or whether the same sample was measured repeatedly
[ ]	[x]	The statistical test(s) used AND whether they are one- or two-sided<br>*Only common tests should be described solely by name; describe more complex techniques in the Methods section.*
[ ]	[x]	A description of all covariates tested
[ ]	[x]	A description of any assumptions or corrections, such as tests of normality and adjustment for multiple comparisons
[ ]	[x]	A full description of the statistical parameters including central tendency (e.g. means) or other basic estimates (e.g. regression coefficient) AND variation (e.g. standard deviation) or associated estimates of uncertainty (e.g. confidence intervals)
[ ]	[x]	For null hypothesis testing, the test statistic (e.g. *F*, *t*, *r*) with confidence intervals, effect sizes, degrees of freedom and *P* value noted<br>*Give P values as exact values whenever suitable.*
[x]	[ ]	For Bayesian analysis, information on the choice of priors and Markov chain Monte Carlo settings
[x]	[ ]	For hierarchical and complex designs, identification of the appropriate level for tests and full reporting of outcomes
[x]	[ ]	Estimates of effect sizes (e.g. Cohen's *d*, Pearson's *r*), indicating how they were calculated

Our web collection on statistics for biologists contains articles on many of the points above.

Software and code

Policy information about availability of computer code

Data collection	Data were recorded on paper and Microsoft Excel spreadsheets.
Data analysis	Data were analyzed using R v. 4.2.1 and will be published in figshare with DOI 10.6084/m9.figshare.25725594 before resubmission. The car v. 3.1-1, MuMIn v. 1.47.1, stats v. 4.2.1 and FSA v. 0.9.5 packages were also implemented.

For manuscripts utilizing custom algorithms or software that are central to the research but not yet described in published literature, software must be made available to editors and reviewers. We strongly encourage code deposition in a community repository (e.g. GitHub). See the Nature Portfolio guidelines for submitting code & software for further information.

Data

Policy information about availability of data

All manuscripts must include a data availability statement. This statement should provide the following information, where applicable:

- Accession codes, unique identifiers, or web links for publicly available datasets
- A description of any restrictions on data availability
- For clinical datasets or third party data, please ensure that the statement adheres to our policy

Data will be available through figshare before resubmission using DOI 10.6084/m9.figshare.25725594.

Research involving human participants, their data, or biological material

Policy information about studies with human participants or human data. See also policy information about sex, gender (identity/presentation), and sexual orientation and race, ethnicity and racism.

Reporting on sex and gender	*Use the terms sex (biological attribute) and gender (shaped by social and cultural circumstances) carefully in order to avoid confusing both terms. Indicate if findings apply to only one sex or gender; describe whether sex and gender were considered in study design; whether sex and/or gender was determined based on self-reporting or assigned and methods used. Provide in the source data disaggregated sex and gender data, where this information has been collected, and if consent has been obtained for sharing of individual-level data; provide overall numbers in this Reporting Summary. Please state if this information has not been collected. Report sex- and gender-based analyses where performed, justify reasons for lack of sex- and gender-based analysis.*
Reporting on race, ethnicity, or other socially relevant groupings	*Please specify the socially constructed or socially relevant categorization variable(s) used in your manuscript and explain why they were used. Please note that such variables should not be used as proxies for other socially constructed/relevant variables (for example, race or ethnicity should not be used as a proxy for socioeconomic status). Provide clear definitions of the relevant terms used, how they were provided (by the participants/respondents, the researchers, or third parties), and the method(s) used to classify people into the different categories (e.g. self-report, census or administrative data, social media data, etc.) Please provide details about how you controlled for confounding variables in your analyses.*
Population characteristics	*Describe the covariate-relevant population characteristics of the human research participants (e.g. age, genotypic information, past and current diagnosis and treatment categories). If you filled out the behavioural & social sciences study design questions and have nothing to add here, write "See above."*
Recruitment	*Describe how participants were recruited. Outline any potential self-selection bias or other biases that may be present and how these are likely to impact results.*
Ethics oversight	*Identify the organization(s) that approved the study protocol.*

Note that full information on the approval of the study protocol must also be provided in the manuscript.

Field-specific reporting

Please select the one below that is the best fit for your research. If you are not sure, read the appropriate sections before making your selection.

☐ Life sciences ☐ Behavioural & social sciences ☒ Ecological, evolutionary & environmental sciences

For a reference copy of the document with all sections, see nature.com/documents/nr-reporting-summary-flat.pdf

Ecological, evolutionary & environmental sciences study design

All studies must disclose on these points even when the disclosure is negative.

Study description	To investigate the impact and drivers of carbon and nutrient fluxes from insect folivores we collected foliar (green leaf and litter) nutrient concentration, soil nutrient concentration, and temperature data at 74 plots in 40 broadleaved forest sites worldwide. Using these and estimates of leaf-level herbivory, we calculated annual fluxes of carbon, nitrogen, phosphorus, and silica deposited from insect herbivores. We compared insect-mediated element fluxes to other major sources of nutrients derived from existing models of atmospheric nitrogen and phosphorus and mineral weathered phosphorus. We summarized our findings by comparing insect-mediated element fluxes between biome types (boreal, temperate, and tropical forests) and investigated potential abiotic and biotic drivers of these fluxes.
Research sample	Research samples included passively collected forest leaves, as well as actively collected fresh green leaves and soil cores. Samples were meant to represent herbivory, herbivore-mediated element fluxes, foliar nutrient concentrations and soil nutrient concentrations at the forest stand level.
Sampling strategy	We largely followed that of existing literature on insect-mediated element fluxes (refs. 10 and 25) and sometimes utilized littertraps from existing networks (e.g., ForestGEO) to determine number of littertraps. Relatively undisturbed forest plots and sites are generally difficult to access (see Methods for selection critereia) and were thus opportunistically rather than statistically chosen. The network, however, did purposefully cover nearly the entire range of broadleaved forest types on Earth (ref. 20).<br><br>To obtain community-level estimates of foliar production and leaf litter nutrients we picked up passively collected litter leaves from 9-25 litter traps at each plot. For green leaves, we sampled and pooled 150 or more (to obtain at least 25 g dry weight) green, healthy (no to minimal signs of damage or discoloration) leaves at multiple heights in the canopy from at least ten randomly selected trees. To estimate herbivory, the corresponding author assessed quarterly scans of leaf litter sent by local teams. To characterize soil, we homogenized ten cores (0-15 cm depth) once during the growing season at each plot. Tomst sensors recorded temperature at several of the plots. Remaining temperature and climate data were estimated from local weather stations and existing literature (see Methods). Atmospheric nitrogen and phosphorus and mineral weathered phosphorus values for each plot were estimated from existing literature (refs. 57-58).

Data collection	All samples were collected by local teams comprised of co-authors and/or assistants listed in the Acknowledgements section for one or two years between 2018 to 2021 at regular intervals during the growing season (exceptions noted in Supplementary Data 1). Local teams collected leaf and soil material, scanned leaves, and processed soils (dried) and leaves (dried and weighed) before shipping samples to the corresponding author. Litter leaf weight data were sent to the corresponding author electronically via Excel or Google spreadsheets. The corresponding author then finished processing samples (further ground leaves and soils, estimated leaf area removed from scans first by paper then electronically transfered to Excel spreadsheets) before shipping to Copenhagen University for carbon, nitrogen and phosphorus anaylsis. The corresponding author also prepared all leaf and soil sample digestions for silica analysis which were then analyzed at Lund University. Nutrient data from labs were sent to the corresponding author electronically.
Timing and spatial scale	Samples were primarily collected for one to two years between years 2018 and 2021 to estimate annual element fluxes. Few exceptions (4 plots) to collection year are described in the Methods section and Supplementary Data Table 1. These exceptions included sites whose collections were affected by the COVID-19 pandemic but whose representation were deemed crucial to the global network of forests. Litterfall samples were collected every two weeks (>2000 mm/y precipitation) to one month (other sites) for one to two years depending on local conditions. Leaf litter (entire or half of each trap) was scanned and assessed for herbivory quarterly at each plot. Green leaf samples were collected every peak growing season for deciduous forests and every dry/wet season for evergreen forests. Soils were collected once per year during peak growing season.<br><br>The 74 plots span six continents and nearly all broadleaved forest types. Please see Supplementary Fig. 1 and Supplementary Data 1 for a site map and coordinates for each plot, respectively. To minimize the confounding influence of disturbance history only mature forests (most classified as old-growth or primary) with no visible signs of recent human activity were chosen. Of the 74 forest plots, 68 plots spanned an area of 1 ha each, and 64 plots were situated at least 450 m from major anthropogenic disturbances such as major roads or settlements. We installed 9 to 25 litter traps (0.1-0.5 m2 in area) in each of the 74 plots such that leaf litter traps were 0.3-1.0 m above the surface of the ground and spaced at approximately 20-m intervals (one site exception to interval distance is described in Supplementary Data 1).<br><br>Start/end dates of collection/installation + exceptions:<br>ANKA1 2020-08-05 to 2021-09-02; ANKA2 2020-08-05 to 2021-09-02; ARJE 2019-08 to 2020-08; BAIK1 2019-07 to 2019-10-24; BAIK2 2019-07 to 2019-10-13; BIAL 2019-10 to 2020-11; BSMA 2019-08-02 to 2021-07-27; CAXI 2010-03 to 2011-03; CHBA0768; 2019-07-23 to 2020-10-22; CHBA1009 2019-07-27 to 2020-10-22; CHBA1800 2019-08-07 to 2020-10-25; CHUK 2019-06-13 to 2020-05-08; COOL2/3 2020-08-28 to 2021-07; COOL5/6 2020-08-25 to 2021-07; COOL7 2020-08-25 to 2021-07; COOL8 2020-08-27 to 2021-07; DANUC1 2016-08-29 to 2016-05-23; DANUC2 2016-08-16 to 2016-05-23; LEWI0090 2019-12-19 to 2021-01-28; EKG01 2019-06-03 to 2020-08-03; EKG02 2019-06-02 to 2020-08-05; FERLD 2019-09 to 2019-11; KILI2169 2019-03-11 to 2021-04-05; KILI2459 2019-03-14 to 2021-04-08; KILI2845 2019-03-14 to 2021-04-05; GEGURI 2020-08-13 to 2020-11-05; GESAIR 2020-08-12 to 2020-11-04; GUTI 2019-06-22 to 2021-04-22, due COVID disruptions we combined data from 2019-05 to 2020-12 and 2021-01 to 2021-04; HIRF2400 2020-03-07 to 2021-03-22; HIRF3000 2020-03-07 to 2021-03-22; HIRF5500 2020-03-08 to 2021-03-22; JIAN 2019-08-24 to 2021-07-20; JOYC1 2020-06-29 to 2020-11-23; JOYC2 2020-06-28 to 2020-11-22; JOYC3 2020-06-30 to 2020-11-22; KEVOJES00 2019-08-06 to 2020-10-06; KEVOJES01A 2019-08-08 to 2020-10-08; KEVOJES02 2019-08-07 to 2020-10-06; LAGON1 06/08 to 2020-11-03; LAGON2 06/08 to 2020-11-01; LAGON3 06/08 to 2020-11-01; LEWI0450 2019-12-19 to 2021-01-21; LEWI1000 2019-12-19 to 2021-01-21; LLAO 2019-06-22 to 2021-04-22, due COVID disruptions we combined data from 2019-05 to 2020-12 and 2021-01 to 2021-04; LSAG1 2019-10-04 to 2020-10-02; LSAG3 2019-10-05 to 2020-10-02; LSAG4 2019-10-06 to 2020-10-06; MABE 2019-08-07 to 2020-10-15; MATG0934 2019-10-05 to 2020-10-04; MATG1024 2019-10-05 to 2020-10-05; MATG1116 2019-10-06 to 2020-10-03; MATG1204 2019-10-05 to 2020-10-05; MATG1274 2019-10-05 to 2020-10-05; NERA0800 2019-07-27 to 2020-11-28; NERA1050 2019-07-28 to 2020-11-29; NERA1300 2019-07-31 to 2020-11-30; PRIM 2020-07-15 to 2020-11-01; PUYE1 2019-08-07 to 2021-07-30; PUYE2 2019-08-07 to 2021-07-30; PUYE3 2019-08-07 to 2021-07-30; QUET 2019-06-22 to 2021-04-22, due COVID disruptions we combined data from 2019-05 to 2020-12 and 2021-01 to 2021-04; RHOD 2019-06-13 to 2020-05-08; RUNC 2019-08-02 to 2020-11-01; SAMO 2019-06-22 to 2021-04-22, due COVID disruptions we combined data from 2019-05 to 2020-12 and 2021-01 to 2021-04; SARO 2020-06-25 to 2021-05-27; SHAP 2019-07-24 to 2020-10-18; SOKO 2019-08-14 to 2020-09-28; TORRE 2010-03 to 2011-03; UDZU 2019-04-03 to 2021-03-05, missed 2020-01, 2020-08, 2020-10, 2020-11, 2021-02 leaf litter collections; VANC 2019-07-01 to 2019-11-23; WAYQ 2018-03 to 2020-3; WRIG 2020-06-19 to 2020-10-31; WYTH 2018-10-12 to 2018-11-23; ZOFI 2019-05-22 to 2020-05-22<br><br>Some start/end dates are approximated. Sample collections delayed by a few days due to weather, etc. are not noted above.
Data exclusions	Data excluded included sites whose herbivory rates were determined as outbreak level rather than background level during the course of the study. We used a combination of literature and local expertise to make these determinations. Other sites were dropped due to incomplete data collections as a result of COVID-19 disruptions.
Reproducibility	We used the means between years for sites where samples were taken for two years. Though observations in this study were likely dependent on spatial and temporal context and were thus difficult to reproduce, computational reproducibility can be achieved by using the same datasets, codes and software as this study.
Randomization	To account for potential similarities due to geographical nearness, we considered site to be a random factor in the data analysis. To address a different research question for the same project, some sites consisted of multiple plots that followed natural environmental gradients (74 plots in 40 forest sites). In these cases, plots were nested within sites for data analysis.<br><br>Littertraps that passively collected leaves were installed 20-m from one another usually in grids. In plots where only half of the leaves were assessed for herbivory, littertrap mesh was permanently marked halfway and collectors randomly scanned one half of each trap for subsampling. For example, B bags from all 20 littertraps were scanned for the month of July, A bags in August, etc. Both A and B bags were later consolidated to determine litter dry weight. Multiple soil cores next to each or half of the littertraps (e.g., all even-numbered littertraps) were consolidated by plot. Green leaves from at least ten random trees from at least three canopy heights (high, middle, low) were collected and consolidated for each plot.

Blinding	This was not a clinical study but litter leaves used to estimate herbivory were passively collected in littertraps installed at 20-m intervals.

Did the study involve field work? ☒ Yes ☐ No

Field work, collection and transport

Field conditions	Field conditions varied throughout the year at each of the 74 plots. General climate information for each location such as mean annual temperature and precipitation can be found in Supplementary Data 1.
Location	Descriptions of all 74 forest plots in the global network including location can be found in Supplementary Data 1.
Access & import/export	The project was required to comply to and document all access, collection and import/explort permits as required by the EU Horizon 2020 grant. Copies of permits may still be found at the Lund University Department of Physical Geography economy office or from co-authors.<br><br>Issuing authority, issue date, identifying information:<br>Sweden, Jordbruksverket, 2021-09-02, 6.4.18-01528/2021; Białowieża in Poland, Ministerstwo Środowiska, 2019-06-18, DOP-WPN.436.104.2019.TP; Erdaobaihe in Jilin in China, Changbai Mountain Nature Conservation and Management Center, 2020-04-27; Bhutan, Ministry of Agriculture & Forests, 2021-06-29, NBC/BRD/7/2020-2021/1697; Cooloola in Australia, Department of Environment and Science, 2019-10-24, PTU19-002346; Likoula in Republic of the Congo, Ministry of Environment, 2018-12-04, 2921/MTE-CAB.18; Kilimanjaro in Morrogoro in Tanzania, Tanzania Commission for Science and Technology, 2018-12-19, 2018-459 NA-2005-141; Tanzania National Parks, 2019-02-25, TNP/HQ/C/10/13; San Carlos de Bariloche in Rio Negro in Argentina, National Parks, 2019-04-05, IF-2019-20686188-APN-DRPN#APNAC; Kevo Strict Nature Reserve in the Municipality of Utsjoki in Finland, Metsähallitus, Luontopalvelut (Metsähallitus, Parks and Wildlife Finland), 2019-06-14, MH 2641/2019; Mt. Lewis National Park in Australia, Department of Environment and Science, 2019-10-24, PTU19-002346; Nantahala National Forest in U. S. A., U. S. Forest Service Department of Agriculture Forest Service, 2020-06-26, 2720; Henry Wright Preserve in U. S. A., Highlands-Cashier Land Trust, 2020-06-11; U. S. Department of Agriculture Forest Service in U. S. A., 2019-07-12, WHR65; White River National Forest in U. S. A., U. S. Department of Agriculture Forest Service, 2019-07-01, 0596-0082; Upper Waiakea Forest Reserve in Hawaii in U. S. A., State of Hawaii Department of Land and Natural Resources, 2021-02-15; Hawaii National Park in U. S. A., National Park Service, 2019-05-29, HAVO-2019-SCI-0023; Kohala Forest Reserve in Hawaii in U. S. A., State of Hawaii Department of Land and Natural Resources, 2020-01-15; Napali-Kona Forest Reserve in Kauai in U. S. A., State of Hawaii Department of Land and Natural Resources, 2018-12-24, KPI-2018-88; Laupahoehoe in Hawaii in U. S. A., Hawaii Experimental Tropical Forest, 2019-02-19; Nera reserve (National park Semenic-Cheile Carasului) in Romania, Regia Nationala a Padurilor - Romsilva, Administratia Parcului National Semenic-Cheile Carasului, 2019-07-30, 1313/30.07.2019; Puyehue National Park, Los Lagos Region in Chile, National Forestry Corporation, 2019-2021, 07-2019; Santa Rosa National Park in Costa Rica, Programa de Investigación, Parque Nacional, Santa Rosa, 2021-02-19, R-SINAC-ACG-PI-011-2021; San José in Costa Rica, SINAC-CITES, 2021-08-21, 2021-CR5611/SJ (#S 8543); Georgia, Agency of Protected Areas, 2020/08/04, 1847-0-2-202008041616; Zofin Primeval Forest Reserve in Czech Republic, Agentura ochrany přírody a krajiny České republiky/ Nature Conservation Agency of the Czech Republic, Správa CHKO Blanský les/Administration of PLA Blanský les, 2020-04-20, SR0057/JC/2020-3; Baikal State Natural Biosphere Reserve in the in the Republic of Buryatia in Russia, Federal State Budgetary Institution, 2019-07-18; Ankasa Conservation Area in Ghana, Forestry Commission, 2018-05-22, WD/A.185/VOL.13/19; General Directorate of Sustainable Management of Forestry and Wildlife Heritage, National Forestry and Wildlife Service in Peru; 2017-03-02, 064-2017-SERFOR/DGGSPFFS; Danum Valley in Malaysia, Sabah Biodiversity Council, Research licence JKM/MBs.1000-2/2 JLD.6 (76); Caxiuana National Forest in Brazil, Ministry of Science and Technology, 2008-05, Portaria no. 283.<br><br>Verbal agreements, sites under the management of co-authors, sites under cooperative agreements with co-author institutes, and sites that did not require permits are not listed.
Disturbance	All littertrap installations continue to be used or were removed upon completion of the project. Leaf and soil materials not used in nutrient analyses were returned to their respective sites after weighing. Due to the short-term nature of the project and relatively infrequent visitations, disturbances due to the project were not considered extensive.

Reporting for specific materials, systems and methods

We require information from authors about some types of materials, experimental systems and methods used in many studies. Here, indicate whether each material, system or method listed is relevant to your study. If you are not sure if a list item applies to your research, read the appropriate section before selecting a response.

Materials & experimental systems

n/a	Involved in the study
☒	☐ Antibodies
☒	☐ Eukaryotic cell lines
☒	☐ Palaeontology and archaeology
☒	☐ Animals and other organisms
☒	☐ Clinical data
☒	☐ Dual use research of concern
☒	☐ Plants

Methods

n/a	Involved in the study
☒	☐ ChIP-seq
☒	☐ Flow cytometry
☒	☐ MRI-based neuroimaging

Plants

Seed stocks	NA
Novel plant genotypes	NA
Authentication	NA
